# Can NT-proBNP Levels Be an Early Biomarker of Reduced Left Ventricular Ejection Fraction in Preterm Infants?

**DOI:** 10.3390/children9071002

**Published:** 2022-07-03

**Authors:** Ya-Lan Lin, Yi-Li Hung, Chung-Min Shen, Yung-Chuan Chen, Wu-Shiun Hsieh

**Affiliations:** 1 Department of Pediatrics, Cathay General Hospital, Taipei 106, Taiwan; duckblue92@gmail.com (Y.-L.L.); b82401103@yahoo.com.tw (Y.-L.H.); shen8471@yahoo.com.tw (C.-M.S.); vvv9496@yahoo.com.tw (Y.-C.C.); 2 School of Medicine, Fu Jen Catholic University, New Taipei City 24205, Taiwan; 3 School of Medicine, National Tsing-Hua University, Hsinchu 30013, Taiwan; 4 Department of Pediatrics, National Taiwan University Children’s Hospital, Taipei 100, Taiwan; 5 Department of Pediatrics, College of Medicine, National Taiwan University, Taipei 100, Taiwan

**Keywords:** brain natriuretic peptide, biomarker, ejection fraction, N-terminal pro-B-type natriuretic peptide, preterm infants

## Abstract

Background/Objective: N-terminal pro-B-type natriuretic peptide (NT-proBNP) is a cardiac natriuretic hormone that cardiomyocytes release in response to ventricular stretch. It helps with the diagnosis of heart failure in adults, but this application in preterm infants has rarely been reported. This study aimed to evaluate whether NT-proBNP could be used for the early detection of reduced cardiac ejection fraction in preterm infants and the optimal timing for NT-proBNP assessment. Design/Methods: This prospective, single-center, observational study enrolled all preterm infants with NT-proBNP measurements from October 2014 to February 2022. They underwent echocardiographic examinations within 48 h of the NT-proBNP measurements. Reduced left ventricular ejection fraction was defined as below 60%. Receiver operator characteristic (ROC) curves were generated to assess the optimal NT-proBNP cutoff point for the early prediction of reduced cardiac ejection fraction. Results: A total of 68 preterm infants were enrolled, with a total of 134 NT-proBNP measurements being available for analysis. Reduced left ventricular ejection fraction was present in seven infants (10.3%) due to various underlying diseases. The NT-proBNP cutoff level for detecting reduced left ventricular ejection fraction was 9248 pg/mL, with 71.4% sensitivity and 60.8% specificity; the area under the curve was 0.623 (95% CI: 0.487~0.760). The threshold for the optimal postnatal age for applying NT-proBNP to detect reduced left ventricular ejection fraction was >2 days of life (AUC: 0.682; 95% CI: 0.518~0.845), with 70% sensitivity and 67.1% specificity. Conclusions: Although the NT-proBNP levels declined dramatically after birth, a NT-proBNP serum level of 9248 pg/mL might be helpful for the early detection of reduced ejection fraction in preterm infants, and the optimal age for detection was after 2 days of life.

## 1. Introduction

NT pro-B-type natriuretic peptide (NT-proBNP) is a natriuretic peptide produced by cardiomyocytes. Its precursor is pro B-type natriuretic peptide (pro BNP), a 108-amino acid (aa) protein. ProBNP is enzymatically cleaved to form NT-proBNP, a 76-aa N-terminal peptide, and B-type natriuretic peptide (BNP), a 32-aa peptide [1]. NT-proBNP is the inactive form, while BNP is biologically active. BNP is secreted when a cardiomyocyte senses changes in volume and pressure in the ventricles. BNP has diuretic, natriuretic, and vasodilatory properties [1]. It also antagonizes the renin–angiotensin system, reducing intravascular volume, and thus improving cardiac function [2].

NT-proBNP has a longer plasma half-life of 1–2 h, compared to BNP’s half-life of 15–20 min; consequently, it has a higher plasma concentration [1]. Additionally, in contrast with BNP, all NT-proBNP immunoassays use the same calibration materials and antibodies, providing comparable values across studies [1].

NT-proBNP can be an independent predictor of heart failure in adults [3]. It has been employed in many aspects of heart disease, such as left ventricular systolic and diastolic dysfunction, ischemic heart disease, and hypertrophic cardiomyopathy [4]. However, few studies have evaluated the changes in NT-proBNP in infants with heart failure, especially in term and preterm neonates. This study aimed to assess whether NT-proBNP could be a useful biomarker for the early identification of preterm neonates with reduced cardiac ejection fraction under conditions with cardiopulmonary impairment and aimed to evaluate the optimal timing for detection.

## 2. Materials and Methods

This was a prospective observational study that recruited preterm infants born at less than 37 weeks gestation and admitted to the Neonatal Intensive Care Unit of Cathay General Hospital from October 2014 to February 2022. The inclusion criteria were all preterm infants with available NT-proBNP results who underwent echocardiographic examinations within 48 h after admission for blood sampling including NT-proBNP measurements. The study excluded patients with major congenital malformations or those without available NT-proBNP measurements. Ethical approval for the data collection was granted by the Ethics Committee of the Cathay General Hospital Taipei, Taiwan (No. CGH-P110091/24 December 2022).

NT-proBNP measurements were obtained for premature infants with clinical suspicion of altered cardiac function, such as congenital heart disease, *patent ductus arteriosus*, heart failure, pulmonary hypertension, or perinatal asphyxia. Repeated measurements were performed when the clinical condition had changed or at follow-up after the treatment of heart failure. Blood samples were collected using ethylenediaminetetraacetic acid tubes and processed immediately. The NT-proBNP levels were measured using an electroluminescence immunoassay kit with the Elecsys proBNP II test (ECLIA^®^, Roche Diagnostics (Basel, Switzerland). The test has detection limits of 5–35,000 pg/mL. All the patients in the study underwent Doppler echocardiography performed by an independent cardiologist blinded to the NT-proBNP study, by whom the left ventricular ejection fraction (LVEF) was assessed. The modified Quinones method was used to estimate the left ventricular cavity size from the M-mode study. The LVEF was the stroke volume/end-diastolic volume × 100. A Philips Ultrasound System iE33 × MATRIX with a neonatal probe was used for each study. A reduced ejection function was defined as an LVEF < 60% [5,6]. Hemodynamically significant *patent ductus arteriosus (hsPDA)* was diagnosed as left-to-right shunting across the PDA associated with a left atrial-to-aortic root ratio > 1.4, and/or left pulmonary artery end-diastolic flow velocity > 0.2 m/s, and/or retrograde diastolic blood flow in the descending aorta [7].

Statistical analysis. Qualitative data were presented as frequencies and percentages and were compared using Fisher’s exact tests or chi-square tests. Quantitative variables were presented as means and standard deviations or medians and ranges according to their distributions. Mann–Whitney U Wilcoxon tests or Student’s t-tests were applied after normality tests were processed in the bivariate analysis of the quantitative variables. Correlation analyses were performed to determine the relationship between NT-proBNP levels and the patient’s age.

To determine an appropriate cut-off value of plasma NT-proBNP for diagnosing reduced ejection fraction in preterm infants, the area under the receiver operating characteristic (ROC) curve, sensitivity, specificity, positive likelihood ratio, and negative likelihood ratio were analyzed. An age threshold for using NT-proBNP for early detection was also explored. Statistical analyses were performed with SPSS version 17.0 (SPSS, Chicago, IL, USA). A *p* value of less than 0.05 was considered statistically significant.

## 3. Results

The study enrolled 68 preterm infants with 134 NT-proBNP measurements. The mean gestational age was 30.8 ± 3.3 weeks (range: 23–36 weeks). The mean number of times that blood samples were taken per patient was 1.97 times (range: 1–7 times; median: 1 time). The timepoint for collecting the samples ranged from 0 to 146 postnatal days (median: 7 days). There was no difference in gestational age between infants with and without reduced ejection function, and there was also no difference in birth body weight between these two groups (Table 1).

Among the 68 enrolled preterm infants, thirty-six (52.9%) had a patent ductus arteriosus (PDA), thirteen patients (19.1%) had congenital heart disease, five patients (7.4%) had severe respiratory disease, four patients (5.9%) had proven sepsis, and the others were included due to necrotizing enterocolitis, hypovolemic shock, neuromuscular disease, infection events, or perinatal asphyxia, etc. There were seven patients (11.1%) with reduced ejection fraction, the causes of which were cardiomyopathy, Pompe disease, ventricular septal defect, perinatal asphyxia, infective endocarditis, the recipient of twin-to-twin transfusion syndrome, and sepsis. The median LVEF of all the patients was 71.00% (44~89%) (Table 1).

**Figure 1:**
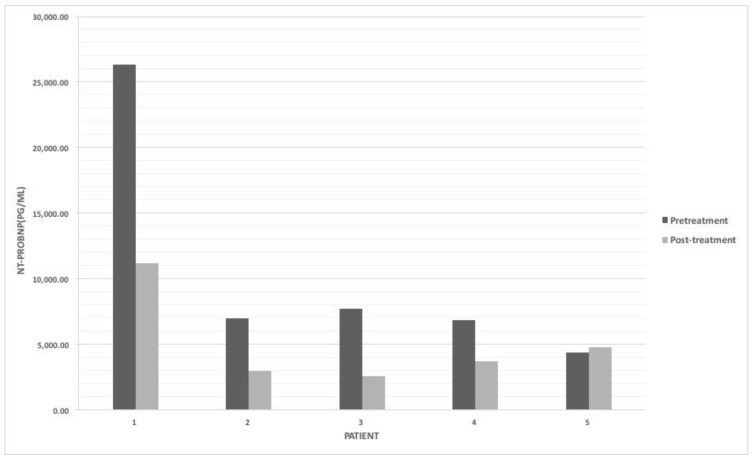
NT-proBNP levels before and after treatment with ibuprofen for patients with hemodynamically significant patent ductus arteriosus.

Among the 36 patients with PDA, 18 had received treatment for PDA closure, 1 of them had undergone surgical ligation, and others had received ibuprofen treatment. Only one patient with PDA had reduced ejection fraction, but this patient also presented with cardiomyopathy. Most of the neonates with PDA in this study did not have reduced ejection fraction (Table 1). The median NT-proBNP level in the patients with PDA was significantly higher than that in patients without PDA (9233.5 pg/mL vs. 4262.5 pg/mL; *p*= 0.003, Mann–Whitney U test). Among the patients who received PDA treatment, five had serial NT-proBNP measurements before and after receiving treatment (Figure 1), and three (Patients 1 to 3) had successful responses. We found that patients with successful responses showed reductions of over 50% in NT-proBNP levels.

Among the thirteen patients with congenital heart disease, eight had ventricular septal defects, two had hypertrophic cardiomyopathy, one had coarctation of the aorta, one had an atrial septal defect, and one had dilated cardiomyopathy. Among them, four patients had reduced left ventricular ejection fraction including congenital hypertrophic cardiomyopathy (two patients), dilated cardiomyopathy (one patient), and a ventricular septal defect (one patient). The median NT-proBNP level was higher in the patients with congenital heart disease than in the patients without congenital heart disease, but this was not statistically significant (13,147.5 pg/mL vs. 6567.0 pg/mL; *p* = 0.05, Mann–Whitney U test).

**Figure 2:**
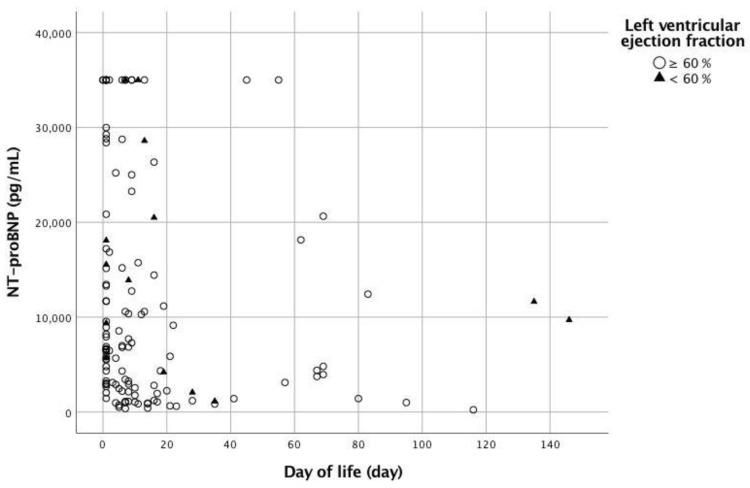
Correlation between N-terminal pro-B-type natriuretic peptide (NT-proBNP) level and age.

The NT-proBNP levels had a negative correlation with the age of the patients without reduced left ventricular ejection function (r_s_ = −0.308, *p* = 0.001); however, there was no negative correlation in the patients with reduced left ventricular ejection function (r_s_ = −0.327, *p* = 0.254, Spearman’s rank sum test) (Figure 2).

**Figure 3:**
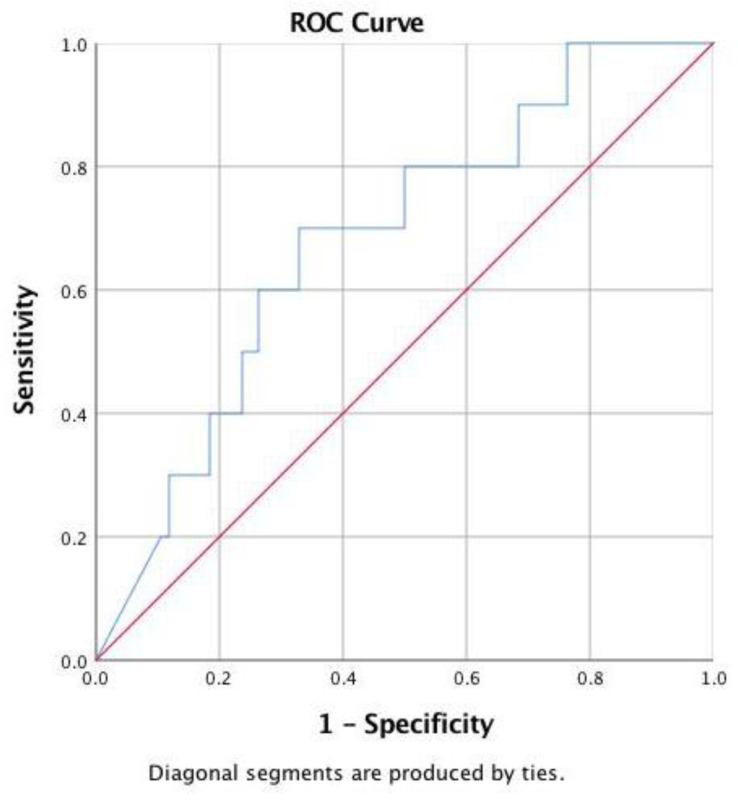
Receiver operator characteristic (ROC) curve for N-terminal pro-B-type natriuretic peptide detecting reduced left ventricular ejection fraction at >2 days. The blue line is the receiver operating characteristic curve. The red line is the diagonal reference line.

The optimal NT-proBNP cutoff value for detecting reduced ejection fraction was 9248 pg/mL (sensitivity: 71.4%: specificity, 60.8%; area under the curve (AUC): 0.623; 95% CI: 0.487~0.760; positive likelihood ratio: 1.82; negative likelihood ratio: 0.47). Because the NT-proBNP level was negatively correlated with postnatal age among the patients without reduced left ventricular ejection fraction, a different ROC curve was used for different postnatal days. A NT-proBNP level > 9421 pg/mL after 2 days of age had the most discriminatory power for detecting reduced left ventricular ejection fraction (sensitivity: 70%; specificity: 67.1%; AUC: 0.682; 95% CI: 0.518~0.845; positive likelihood ratio: 2.12; negative likelihood ratio: 0.45) (Table 2, Figure 3). 

## 4. Discussion

A previous study had noted that the levels of NT-proBNP were the highest in the first days of life in full-term infants and showed a marked decline during the first week of life. They noted that the reference values of NT-proBNP in infants and children varied across wide ranges, 260–13,224 pg/mL and 28–7250 pg/mL, during days 0–2 and 3–11 of life, respectively, and found that the level dropped dramatically after 3 days of life [4]. Harris et al. and Fritz et al. reported a similar phenomenon in preterm infants [8,9]. In this study, we also found that, in preterm infants without reduced cardiac ejection fraction, the NT-proBNP levels declined gradually after birth. Increased left and right ventricular pressure and volume overload after birth stimulate the production of natriuretic peptides. The decrease in NT-proBNP levels, starting at 3 days of life, is probably related to the physiologic decrease in pulmonary arterial pressure and diuretic phase that begins shortly after birth. The extremely high NT-proBNP levels at birth might reflect the response to the intrauterine cardiovascular conditions. One study noted that fetal distress, a younger gestational age at birth, multiple pregnancies, antenatal magnesium sulfate, and intrauterine growth restriction were significantly associated with elevated BNP levels in neonates [10]. Harris et al. noted that early NT-proBNP levels were higher in infants who developed severe bronchopulmonary dysplasia and in those with renal function impairment [8]. These levels were not affected by hemoglobin levels, recent exposure to hypoxia, or gestational age [8]. Furthermore, the failure of the respiratory and cardiovascular systems to adapt to extrauterine life may lead to persistent pulmonary hypertension, as some studies noted a correlation between persistent pulmonary hypertension and BNP or NT-proBNP levels [11]. Reynold et al. found elevated BNP levels in infants with persistent pulmonary hypertension but not in infants with other forms of respiratory distress [11]. They also found that there was a statistically significant correlation between BNP and tricuspid regurgitation [11]. Shah et al. further reported that BNP levels > 30 ng/dL could predict the requirement for iNO in infants with persistent pulmonary hypertension with 100% sensitivity and 80% specificity [12]. Hilgendorff et al. suggested that NT-proBNP could be used to track the clinical statuses of infants with suspected pulmonary hypertension [13]. 

The pulmonary pressure is usually higher among preterm infants than term infants, which might be due to the delayed occurrence of the diuretic phase. Some studies had noted that the NT-proBNP levels were higher in preterm infants than in full-term infants [12]. In our study, when considering NT-proBNP as a biomarker for predicting reduced cardiac ejection function in preterm infants, the sensitivity and specificity increased after 2 days of life, which could be because this is when the cardiac function had the greatest effect on postnatal NT-proBNP levels. The influence of the intrauterine conditions decreased, and renal function improved 2 days after birth. Therefore, using NT-proBNP as a tool to detect reduced ejection function might be very effective. Nir et al. reported that the 97.5th percentile for NT-proBNP in normal infants at 3–11 days of age was 6502 pg/mL [4]. We found that NT-proBNP levels > 9248 pg/mL could predict reduced cardiac ejection function in preterm infants with a fair level of sensitivity but with unsatisfactory specificity. 

In our study, the LVEF, measured via echocardiography, was used to define reduced ejection fraction, so the method of determining the LVEF could have influenced the diagnosis of reduced ejection fraction. The modified Quinones method was used, which is less precise for tracing the left ventricular cavity than the modified Simpson method [14]. However, in this study, only one pediatric cardiologist performed all of the echocardiograms in order to decrease the inter-operator bias. Heart failure in preterm infants could occur with high cardiac output or with low cardiac output. We focused on low cardiac output in this study, and we evaluated the patients with reduced LVEF to assess if NT-proBNP was a good detection biomarker. However, there remained a group of heart failure patients with normal or high LVEF, which was a limitation in this study. 

Some studies had used measures of the infant’s clinical condition, such as the modified Ross score or the NYHA classification, to define heart failure [1]. Lin et al. reported that, in heart failure patients aged 0–1 years, the optimal cutoff value for NT-proBNP for diagnosing heart failure was 502 pg/mL, resulting in a ROC of 0.795 [3]. They used the modified Ross score to select heart failure patients, but the age range for the development of heart failure was too large for application in preterm infants [3]. Iacob et al. reported that NT-proBNP levels correlated with the severity of heart failure in patients younger than 3 years, but their results were not statistically significant [15]. 

One of the primary causes of heart failure in premature infants is congenital heart disease. In this study, patients with congenital heart disease had significantly higher NT-proBNP levels than patients without congenital heart disease. This was also noted in a previous study using BNP levels to diagnose congenital heart disease in the first month of life, where the test’s diagnostic accuracy was the lowest in the first 3 days of life [16]. One prospective study noted that, in infants with congenital heart disease and with a left-to-right shunt, a NT-proBNP level > 10,000 pg/mL at 7–14 days of life was a useful predictor of early surgery [17]. Changes in BNP levels had been reported as an early predictor of outcomes following surgical repair [18].

NT-proBNP levels can also be influenced by several extracardiac conditions such as pulmonary disease, endocrine or metabolic disorders, liver cirrhosis with ascites, renal failure, inflammatory diseases, cardiotoxic drugs, anemia, obesity, severe infection, and cardiac trauma [1]. Therefore, all aspects of the preterm infant’s clinical condition must be considered when assessing the utility of NT-proBNP. In our study, 36 patients had PDA, and 18 had undergone treatment. The median NT-proBNP level in patients with PDA was significantly higher than that in those without PDA. One systemic review reported that the sensitivity and specificity of using NT-proBNP to diagnose a significant PDA was 90% and 84%, respectively [19]. However, they stated that it was impossible to generalize these results due to the differences between centers in the assay methods and patient characteristics [19]. One study noted that an NT-proBNP level > 10,000 pg/mL at 48 h of age yielded a 100% positive and 87% negative predictive value for excluding spontaneous ductal closure [20]. An early and low NT-proBNP value was a reliable, independent marker for predicting spontaneous ductal closure in preterm infants [17]. Hsu et al. noted that a baseline BNP level > 1805 pg/mL had 88% sensitivity and 87% specificity for predicting indomethacin non-responsive PDA [21]. Ding et al. found that preterm infants who received oral ibuprofen for PDA treatment had significantly decreased NT-proBNP levels at 3 and 7 days after treatment, compared with a placebo group [22]. Hammerman et al. found that the post-treatment NT-proBNP levels in the non-responders remained significantly higher than those in the responders, but the post-treatment/pretreatment ratio was not well correlated with the ductal therapeutic outcome [23]. Therefore, NT-proBNP might not be a good index for diagnosing PDA, but it could be used as a biomarker to predict the treatment response of hemodynamically significant PDA in neonates. In our study, we found that the level of decrease in NT-proBNP before and after the treatment of PDA might predict the effectiveness of the treatment. A decrease in the level of NT-proBNP of more than 50% might predict a favorable outcome. However, our case numbers were limited; a larger-scale study will be crucial for investigating the correlation between the attenuation of NT-proBNP and the closure of hs-PDA.

In conclusion, our study demonstrated that an NT-proBNP serum level of 9248 pg/mL might be helpful for the early detection of reduced left ventricular ejection fraction in preterm infants, but the discrimination power was only fair. From our observation, it was difficult to use NT-proBNP to predict heart failure in preterm infants because it could remain high during the first two days of life. In addition, multiple other factors could also influence the NT-proBNP levels in preterm infants including cardiovascular conditions, fetal distress, pulmonary hypertension, respiratory status, PDA, and renal function. Furthermore, our total number of cases is limited and as there are small numbers of reduced left ventricular ejection fraction in our study, more large-scale studies in the future are needed to make the cut-off values much more precise.

## Figures and Tables

**Table 1:** Demographic data and echocardiogram results.

	Total N = 68, M = 134	EF < 60% N = 7, M = 14	EF ≥ 60% N = 61, M = 120	*p* Value
Median gestational age (weeks)	30.00 (23~36)	30.00 (27~36)	30.50 (23~36)	0.327
Median birth weight (gm)	1360 (540~2940)	1158 (751~2820)	1387.5 (540~2940)	0.068
Patients with PDA	N = 36, M = 64	N = 1, M = 1	N = 35, M = 63	0.001
Patients with congenital heart disease	N = 13, M = 24	N = 4, M = 6	N = 9, M = 18	0.002
Median left ventricular ejection fraction (%)	71.00 (44~89)	52.5 (44~59)	72.00 (60~89)	0.000
Median proBNP (pg/mL)	6833.00 (230–35,000)	12,763 (1129–35,000)	6578 (230–35,000)	0.132

N—number of patients; M—numbers of measurements; EF—ejection fraction. PDA—*patent ductus arteriosus*; NT-proBNP—N-terminal pro-B-type natriuretic peptide.

**Table 2:** Age threshold for detecting reduced ejection fraction.

Age Threshold (Days)	Area under the Curve	NT-proBNP (pg/mL)	Sensitivity (%)	Specificity (%)	Likelihood Ratio (+)	Likelihood Ratio (−)
All ages	0.623	9248	71.4	60.8	1.82	0.47
>1	0.671	9421	70	65.8	2.05	0.46
>2	0.682	9421	70	67.1	2.12	0.45
>3	0.680	9421	70	66.7	2.10	0.45
>4	0.679	9421	70	66.2	2.07	0.45
>5	0.667	9421	70	64.2	1.96	0.45

## Data Availability

The datasets generated and analyzed during this study are available from the corresponding author on reasonable request. hsiehws@ntu.edu.tw.
